# Is Homosexuality a Paraphilia? The Evidence For and Against

**DOI:** 10.1007/s10508-012-9900-3

**Published:** 2012-01-27

**Authors:** James M. Cantor

**Affiliations:** 1 Sexual Behaviours Clinic, Law and Mental Health Program, Centre for Addiction and Mental Health, 250 College Street, Toronto, ON M5T 1R8 Canada; 2 Department of Psychiatry, University of Toronto, Toronto, ON Canada

**Keywords:** Fraternal birth order, Handedness, Neuroanatomy, Neuropsychology, Physical height, Sexual orientation

## Abstract

Whether homosexuality should be described as one among many paraphilic sexual interests or an altogether different dimension of sexual interest has long been discussed in terms of its political and social implications. The present article examined the question instead by comparing the major correlates and other features of homosexuality and of the paraphilias, including prevalence, sex ratio, onset and course, fraternal birth order, physical height, handedness, IQ and cognitive neuropsychological profile, and neuroanatomy. Although those literatures remain underdeveloped, the existing findings thus far suggest that homosexuality has a pattern of correlates largely, but not entirely, distinct from that identified among the paraphilias. At least, if homosexuality were deemed a paraphilia, it would be relatively unique among them, taxonometrically speaking.

## Introduction

Is homosexuality a paraphilia? In the science of sexology, this is a fundamental question for understanding human sexual interests; however, there also exist authors interested in the question because of perceived political implications. Atypical sexual interests remain highly stigmatized in Western society, especially in the United States. Homosexuality, more than any other atypical sexual interest, has achieved greater social acceptance over time, and advocates for other atypical sexual interests—BDSM, cross-dressing, diaperism, etc.—understandably seek the same recognition and rights. Thus, thinking of paraphilias as merely another sexual orientation suggests the conclusion that *everyone* with an atypical sexual interest should benefit from greater tolerance. (Conversely, there exist groups, typically conservative religious groups, who claim that only mainstream, non-paraphilic heterosexuality is acceptable, making any distinctions among other sexual interests entirely moot.)

It is here that I must draw an important, but usually unmarked, distinction: I personally agree wholeheartedly that everyone with atypical sexual interests deserves respect and full recognition of all their civil rights; however, I disagree that answers to scientific questions can be identified by presuming the desired outcome and then backwards-engineering one’s interpretation of the research data to guarantee arrival at that outcome. Moreover, and perhaps more importantly, questions of rights fall outside the purview of science. People deserve respect and civil rights *regardless* of the scientific classification of their sexual interests.

Whether homosexuality is a paraphilia can be addressed by defining those terms as desired. That is, because the field has not yet identified any objective, observable characteristic by which to draw the line between sexual interests that are paraphilic and those that might be called *euphilic* (i.e., non-paraphilic), one can reasonably compose either broader or narrower definitions. For example, paraphilias might reasonably be defined as sexual interests that are atypical for one’s species or, alternatively, as sexual interests that are atypical for one’s sex. The former would exclude homosexuality as a paraphilia, and the latter would include it.

Another (and perhaps a more fruitful) approach to the question is to compare the various correlates and associated features of homosexuality with those of the acknowledged paraphilias. If when considering these various features—prevalence, sex ratio, onset and course, fraternal birth order, physical height, handedness, IQ and cognitive neuropsychological profile, and neuroanatomy—homosexuality falls within the range typical of the paraphilias, then one would more reasonably deem homosexuality another member of that same family. If, however, homosexuality repeatedly lacks or falls at the extremes of the range of those features, then homosexuality would more logically be classified as distinct from the paraphilias.

## Terminology

Many overlapping (and often inconsistent) terms have been used in describing atypical sexual interests and many terms that seem clear within one context are ambiguous in another. Because the present article spans several literatures and multiple contexts, the terms appearing in the cited literature are largely replaced with the following terms and definitions. Although some phrases become wordier, the increased precision they provide is of greater importance for the present purpose.

### Heterosexuality

Predominant sexual interest in the opposite sex. For emphasis, the term applies both to interest in adults as well as to children of the opposite sex—Men sexually interested in adult women and men sexually interested in prepubescent girls are both heterosexual. This usage contrasts with many common contexts, in which the interest in adults is presumed.

### Homosexuality

Predominant sexual interest in persons of the same sex. As with heterosexuality, the term applies both to interest in adults and children of the same sex.

### Pedohebephilia

Predominant sexual interest in children (of either sex), either prepubescent (typically under age 11), early pubescent (typically ages 11–14), or both. Many authors have historically applied the term “pedophilia” regardless of whether the sexually preferred children were pubescent or prepubescent. More recently, increased precision has been sought by restricting “pedophilia” to refer only to the interest in prepubescent children, “hebephilia” for that in early pubescent children, and pedohebephilia as an umbrella term (Blanchard, 2010).

### Teleiophilia

Predominant sexual interest in adults (of either sex) (Blanchard et al., 2000).

### Androphilia

Predominant sexual interest in adult males. Thus, a gay man is an androphilic male and most women are androphilic females.

### Gynephilia

Predominant sexual interest in adult females. Thus, the great majority of men are gynephilic males and lesbians are gynephilic females.

### Euphilia

As noted already, euphilia refers to typical, as opposed to paraphilic, sexual interests. Thus, the question being addressed in this article is whether homosexual persons are euphilic.

The scientific study of correlates and other associated features of sexual interests is very incomplete. Although some findings have been pursued and replicated by multiple investigators, many have not. Thus, the data and their implications must be deemed preliminary at best. Also, researchers did not choose which features to investigate in order to answer the question being pursued here. Rather, they were chosen to answer theoretical questions within their own contexts. That is, there undoubtedly exist other, still unexplored features that could eventually reverse the pattern revealed by the data currently at our disposal.

## Prevalence

The prevalence of atypical sexual behaviors is notoriously difficult to estimate—some extraneous factors can inflate estimates, whereas others can deflate them. Because the stigma of homosexuality and the paraphilias would reasonably reduce the number of people who admit to them, and because Western societies have become much more accepting of homosexuality than of the paraphilias, one cannot discern to what extent differences in reported prevalence might reflect differences in stigma rather than in genuine frequencies. Conversely, there exist people who engage in sexual behaviors outside their genuine, enduring sexual preferences: Same-sex sexual behavior (especially during puberty and adolescence) does not always reflect an underlying preference for same-sex partners over opposite-sex partners, and engaging in exhibitionism, for example, does not always reflect an enduring, underlying preference for exhibitionism over coitus.

Methodological issues notwithstanding, high quality surveys have generally reported a prevalence of enduring homosexuality in approximately 2–4% of the population of Western countries (e.g., ACSF Investigators, 1992; Billy, Tanfer, Grady, & Klepinger, 1993; Chandra, Mosher, Copen, & Sionean, 2011; Dickson, Paul, & Herbison, 2003; Fay, Turner, Klassen, & Gagnon, 1989; Johnson, Wadsworth, Wellings, Bradshaw, & Field, 1992; Laumann, Gagnon, Michael, & Michaels, 1994). Estimates vary, with survey questions containing limits such as “…ever in your life” producing higher estimates than “…since age 18,” and limits such as “…in the past five years” producing higher estimates than “…in the past year.” Although some activist groups and media outlets periodically claim that 10% of the general population is homosexual (for a critical review, see Pruitt, 2002), that quantity did not result from any meaningfully representative sample of the general population (Diamond, 1993; Laumann et al., 1994).

The prevalence of the paraphilias is virtually unknown and might reasonably be called unknowable, given the many practical difficulties in making such an estimate. Some attempts have been made to estimate the frequency of some paraphilic behaviors, but these must be interpreted very cautiously: As already noted, some unknown proportion of people who engage in any given sexual behavior do so for reasons other than to express a genuine sexual preference.

In a representative survey of 18–60 year-olds in the general population of Sweden, 1.67% of the overall sample responded affirmatively to “Have you ever dressed in clothes pertaining to the opposite sex and become sexually aroused by this?” (Långström & Zucker, 2005). It is not known what proportion of these persons would also have endorsed regularly engaging in cross-dressing for sexual arousal, however. In the same survey, 3.1% answered “yes” to the question “Have you ever exposed your genitals to a stranger and become sexually aroused by this?” (Långström & Seto, 2006). Of them, 23.7% reported experiencing sexual fantasies about engaging in the behavior. Of the whole sample, 7.8% answered “yes” to “Have you ever spied on what other people are doing sexually and become sexually aroused by this?” (Långström & Seto, 2006). Of them, 53.4% reported also experiencing sexual fantasies about engaging in that behavior.

Thus, overall, the number of people who engage in homosexual behavior at some point during life may approximate the number of people who engage in a seemingly paraphilic behavior at some point during life, but the (lower) number of people who genuinely and enduringly prefer homosexuality to heterosexuality cannot, as yet, be meaningfully compared to the number of people who genuinely and enduringly prefer one or more paraphilic expressions to non-paraphilic ones.

## Sex Ratio

Homosexuality has been shown repeatedly to occur more frequently among men than in women: 1.4% versus 0.4% for past 5 years in ACSF Investigators (1992); 1.2% versus 0.8% in Dickson et al. (2003); 4.1% versus 2.2% for past 5 years in Laumann et al. (1994); and 6.2% versus 3.6% (United States), 4.5% versus 2.1% (United Kingdom), and 10.7% versus 3.3% (France) since age 15 in Sell, Wells, and Wypij (1995). Interestingly, in men, homosexuality is more common than is bisexuality whereas, in women, bisexuality is more common than is homosexuality (e.g., Chandra et al., 2011; Egan, Edelman, & Sherrill, 2008).

In contrast with the approximately 2:1 ratio of homosexuality in men versus women, paraphilia appears to be a phenomenon exclusive to males, with only very few exceptions. Although no meaningful census can be conducted for paraphilic individuals, neither clinics, forensic institutions, nor social clubs for paraphilic enthusiasts report any substantial number of female paraphilics. Sexual masochism appears to be unique among the paraphilias in the relative frequency of female practitioners: Breslow, Evans, and Langley (1985) surveyed subscribers to and advertisers in a periodical catering to individuals interested in masochism or hyperdominance. Of the 81 non-prostitutes who preferred or usually preferred masochistic behaviors (termed “submissive” in the survey), 49.4% were women. Similarly, Ernulf and Innala (1995) analyzed messages on an on-line discussion group catering to people with the same interests: Of the 56 posts seeking to engage in masochistic acts (again termed “submissive”), 58.9% were from women.

Interestingly, the women who report having paraphilic interests also report homosexual interests much more frequently than do women in general. This invites the interesting speculation that whatever neurodevelopmental processes masculinize an otherwise female brain to manifest male-typical sexual interests may also predispose it to male-typical sexual disorders.

The very large difference in the sex ratios of homosexuality versus any paraphilia suggests that homosexuality and the paraphilias are distinct phenomena; however, there is at least one other plausible interpretation. Although male homosexuality seems an obvious analogy to female homosexuality, there is actually no basis at all for asserting that homosexual men are homosexual for the same reasons that homosexual women are homosexual: Male homosexuality is associated with an entirely different set of correlates and (therefore) etiological contributors from female homosexuality. It is therefore possible that *male* homosexuality is a paraphilia, whereas female homosexuality is not.

## Onset and Course

Other than by being sexual, the most salient feature on which male homosexuality and the paraphilias resemble each other is their lifelong nature—starting in childhood and being immutable despite all efforts to convert them to conventional sexual interests. There have periodically been claims of successful conversion of homosexuality to heterosexuality (e.g., Spitzer, 2003) or of paraphilia to euphilia (e.g., Fedoroff, 1992), but such observations are perhaps better attributed to more mundane reasons, such as demand characteristics, suppression of only the overt expression of the undesired behavior(s), or a reduction of sexual desire in general, rather than in any change in actual focus of whichever sexual interest. Similarly, reports of adult-onset paraphilias (e.g., Mendez, Chow, Ringman, Twitchell, & Hinkin, 2000) might instead be attributed to (typically neuropathological or drug-induced) loss of the ability to suppress already-existing interests.

The research literature supports the childhood onset for a wide range of paraphilias, including rubber fetishism (Gosselin & Wilson, 1980), cross-dressing (Brown et al., 1996), apotemnophilia (First, 2005), acrotomophilia (Dixon, 1983), homosexual or bisexual foot and shoe fetishism (Weinberg, Williams, & Calhan, 1995), and masochism and hyperdominance (Breslow et al., 1985). Interestingly, many paraphilics recall events from early childhood during which they became and then remained fascinated with the object(s) or behaviors of their future sexual interest (e.g., Denko, 1973; Dixon, 1983; Freund, Seto, & Kuban, 1995; Gorman, 1964; Massie & Szajnberg, 1997; Vanden Bergh & Kelly, 1964; Weinberg, Williams, & Calhan, 1994; Weinberg et al. 1995). In homo-/heterosexuality, awareness (and memory) of sexual attractions also begin in childhood, typically before age 10, accompanying maturation of the adrenal glands rather than the gonads (Herdt & McClintock, 2000; McClintock & Herdt, 1996).

## Fraternal Birth Order (or “Older Brother”) Effect

One of the most replicated observations in male homosexuality research is that, among all the children born to a woman, the later born males are more likely to be homosexual than are the earlier born males (Blanchard, 1997, 2004, 2008; Cantor, Blanchard, Paterson, & Bogaert, 2002). In other words, homosexual men have more older brothers, on average, than do heterosexual men. The number of younger brothers has no consistent effect; neither older nor younger sisters has any consistent effect; and there does not appear to be any association between female homosexuality and birth order of any type. Blanchard hypothesized that the fraternal birth order effect on male homosexuality was caused by the immune system of the mother, which becomes increasingly sensitized to proteins produced by the Y-chromosome of each succeeding male fetus and increasingly likely to effect the sexual differentiation of each succeeding male fetus (Blanchard, 2001; Blanchard & Klassen, 1997). Because a female fetus has no Y-chromosome and produces no such proteins, the progressive sensitization occurs during the gestation of only a male fetus. That hypothesis would predict that the fraternal birth order effect would fail to appear among adopted siblings: That is, homosexuality would relate to a child’s position only among his biological siblings and not his position among adopted siblings (e.g., Lalumière, Harris, Quinsey, & Rice, 1998). That prediction has subsequently been borne out in a large-scale study of adopted children (Bogaert, 2006).

Within the paraphilias, fraternal birth order has been examined mostly in pedohebephilia and autogynephilia (a male’s sexual interest in himself in a female or feminized form; Blanchard, 1989a, 1991), but also in exhibitionism and transvestism. None has thus far been associated with a birth order effect. Blanchard et al. (2000) compared four phallometrically assessed groups of men: homosexual pedohebephiles, bisexual pedohebephiles, heterosexual pedohebephiles, and nonoffender heterosexual teleiophiles recruited from the community. No significant difference in fraternal birth order was detected between the heterosexual pedohebephiles and the heterosexual teleiophiles, despite that a significant difference in fraternal birth order was detected between the heterosexual pedohebephiles and the homosexual pedohebephiles. (The presence of the fraternal birth order effect between heterosexual and homosexual pedohebephiles was also reported in Bogaert, Bezeau, Kuban, & Blanchard, 1997.) As noted by Blanchard et al. (2000), these findings suggest that the fraternal birth order effect pertains to homosexuality per se and not to pedophilia.

Information about fraternal birth order in autogynephilia can be gleaned from studies of men with gender dysphoria—biological males who seek surgical sex reassignment and other interventions for bodily feminization. Gender dysphoric males express any of several sexual orientations, including homosexual (relative to their biological sex), heterosexual, bisexual, and asexual (Cantor, Blanchard, & Barbaree, 2009). The latter three types (collectively called the nonhomosexual type) all exhibit autogynephilia (Blanchard, 1989b, 2005) whereas the homosexual type instead exhibits childhood gender nonconformity and other features common among ordinary gay men. The fraternal birth order of autogynephilic versus homosexual gender dysphorics has been compared in samples from Canada (Blanchard & Sheridan, 1992), from the Netherlands (Blanchard, Zucker, Cohen-Kettenis, Gooren, & Bailey, 1996), and from the United Kingdom (Green, 2000). In all three studies, the homosexual males seeking sex reassignment had a significantly greater fraternal birth order than the autogynephilic males seeking sex reassignment. Although none of the three studies included a sample of non-dysphoric men, the magnitude of the groups’ differences was virtually identical to that between ordinary (non-gender dysphoric) homosexual males and ordinary heterosexual males (cf., Green, 2000; Cantor et al., 2002).

Raboch and Raboch (1986) retrospectively examined data from men who attended a Czech sexology clinic between 1955 and 1975. The groups comprised: 249 exhibitionists, 437 pedophilic men (victim age and sex unreported, but presumably prepubescent children of either sex), 57 offenders against males under age eighteen (and presumably pubescent or adolescent), 238 sexual aggressors against women, and 600 men with a sexual dysfunction. Although birth order was not recorded with the precision of the aforementioned studies, the members of the homosexual group were more than twice as likely to be “the last of three or more children” (p. 75) than the members of the comparison group (with sexual dysfunctions rather than atypical sexual interests; 12.2% vs. 28.1%). Neither the exhibitionists (13.7%) nor the sexual aggressors (13.9%) differed markedly from the comparison group. The pedophiles (17.2%) were somewhat intermediate, but it is unknown to what extent this relative elevation might be due to the presence of homosexual pedophiles among the heterosexual pedophiles within the group.

Langevin, Langevin, and Curnoe (2007) provided birth order data on a mostly forensic sample of male paraphiles and sex offenders, which included a group of exhibitionists, of transvestites, and of a heterogeneous “polymorphous” group, none of which differed significantly from the nonoffender control group. Although the analysis in that article provided equivocal results with regard to homosexuality and fraternal birth order, a subsequent re-analysis confirmed the presence of the effect in that dataset (Blanchard, 2007).

Finally, two studies analyzed the fraternal birth orders among samples of heterogeneous sexual offenders (Côté, Earls, & Lalumière, 2002; MacCulloch, Gray, Phillips, Taylor, & MacCulloch, 2004). Although both studies detected a significant fraternal birth order effect, a sizeable portion of the samples had committed offenses against male children. It is therefore unclear whether the fraternal birth order effect emerged because of the homosexuality in the sample rather than because of any other paraphilic interests among the offenders.

Considered together, the fraternal birth order effect appears to be a phenomenon pertaining to male homosexuality, but not the paraphilias.

## Height

In homosexuality research, physical height has been studied to test the sex-reversal hypothesis of homosexuality—that homosexual sexual interest is one result of more generally incomplete sexual dimorphism emerging during development. Multiple studies have been carried out in both small and large samples, but have provided only mixed results. Some studies reported androphilic men to be shorter than gynephilic men, usually 1–2 cm (Bogaert, 2003, 2010; Bogaert & Blanchard, 1996; Martin & Nguyen, 2004). Other studies, however, reported no significant differences, including a re-analysis of the Kinsey database and a large, representative population survey (Blanchard & Bogaert, 1996; Bogaert & Friesen, 2002; Coppen, 1959). There has also been a report in which androphilic men were 1.7 cm taller than their gynephilic counterparts (Evans, 1972). Lesbian women have been reported to be 1.0–1.2 cm taller than androphilic women (Bogaert, 1998; Martin & Nguyen, 2004).

The association of pedophilia and hebephilia with physical height has been studied as part of investigating whether men with those erotic age preferences suffered adverse conditions during childhood or in utero development. Factors such as poor nutrition, pathogen exposure, or economic circumstances retard normal growth and result in lower than average physical height (e.g., Gunnell, 2002; Silventoinen, Lahelma, & Rahkonen, 1999; Wadsworth, Hardy, Paul, Marshall, & Cole, 2002). These studies have revealed a more consistent deficit in height, approximately 1.1–2.0 cm (Cantor et al., 2007; Mellan, Nedoma, & Pondĕlíčková, 1969; Taylor, Myers, Robbins, & Barnard, 1993).

The other paraphilia for which quantitative data have been reported is autogynephilia. These reports compared androphilic (or “homosexual”) male-to-female transsexuals with autogynephilic (or “heterosexual”) transsexuals and found the homosexual type to be substantially shorter than their autogynephilic counterparts (172.9 cm vs. 175.7 cm in Blanchard, Dickey, & Jones, 1995; 173 cm vs. 175 cm in Smith, van Goozen, Kuiper, & Cohen-Kettenis, 2005). Although both studies reported the nearly identical difference in mean height, both studies also reported heights that were substantially shorter, in absolute terms, than the studies that compared non-transsexual homosexual males with non-transsexual heterosexual males. This may reflect sampling error, but it might also reflect (to some extent) a self-selection bias: The participants in the Blanchard et al. (1995) and in Smith et al. (2005) studies were all applying for surgical sex reassignment. It is conceivable that taller persons are less likely to pursue permanent sex reassignment, in the anticipation that their greater height would hinder their ability to be perceived as female. This would leave the remaining surgical applicants to be shorter, on average.

A meta-analysis of height in homosexuality is beyond the scope of the present article, but such a study might provide an interesting and important clarification. Thus, considered collectively, there is evidence that some paraphilias might be deficient in height, but only mixed evidence that male homosexuality is associated with shorter stature.

## Handedness

Handedness reflects brain organization (specifically, cerebral dominance) and offers a window into prenatal brain development. Humans express their handedness prenatally, such as through thumb-sucking in utero (Hepper, Shahidullah, & White, 1991; Hepper, Wells, & Lynch, 2005). Although there exist natural left-handers who may inherit left-handedness genetically, non-right-handedness (which includes both left-handedness and ambidextrousness) can also be caused by perturbations of cerebral development (Bishop, 1990). When one hemisphere of the brain suffers damage during development, the other may take on additional functions, including those expressed through handedness (Bakan, 1971; Bakan, Dibb, & Reed, 1973). In the general population, the frequency of non-right-handedness is approximately 8–15% (Hardyck & Petrinovich, 1977), but populations with any of several neurological disorders express non-right-handedness 1.5–3.0 times more frequently, including Down’s Syndrome (e.g., Batheja & McManus, 1985), epilepsy (e.g., Schacter et al., 1995), autism (e.g., Soper et al., 1986), learning disabilities and dyslexia (e.g., Cornish & McManus, 1996), and mental retardation (e.g., Grouios, Sakadami, Poderi, & Alevriadou, 1999). There is a sex difference in handedness, with the odds of non-right-handedness approximately 25% higher in men than in women, as reported by meta-analytic reviews of the literature (Papadatou-Pastou, Martin, Munafò, & Jones, 2008; Sommer, Aleman, Somers, Boks, & Kahn, 2008).

Handedness has been examined in multiple studies of androphilic men and gynephilic women, which have subsequently been meta-analyzed (Lalumière, Blanchard, & Zucker, 2000): In that quantitative review, the androphilic men (i.e., gay men) showed 34% greater odds of being non-right-handed than did gynephilic men. That is, the androphilic men were shifted more towards the male-typical direction relative to the gynephilic men. The gynephilic women (i.e., lesbian women) showed 91% greater odds of being non-right-handed than did the androphilic women. Interestingly, the results for the androphilic men run counter to what the brain-sex theory of homosexuality would predict: Both homosexual groups showed *more* of the male-typical trait than did straight men. It is interesting to speculate whether *two* prenatal mechanisms might be in play: one being a perturbation that increases non-right-handedness in each group and one that increases sexual atypicality. In lesbian women, both mechanisms would increase the odds of non-right-handedness whereas, in gay men, these mechanisms might partially offset each other.

Rates of non-right-handedness have also been reported in male sexual offenders against children (Bogaert, 2001) and in phallometrically assessed pedophilic men (Cantor et al., 2004; Cantor, Klassen, et al., 2005), with the pedophiles showing approximately 3.5 times greater odds of non-right-handedness than teleiophiles. The size of this effect is in the range reported for the aforementioned pervasive neurodevelopmental disorders (Down’s Syndrome, etc.). Rahman and Symeonides (2008) calculated a “variance quotient” for a convenience sample of men on the basis of their responses to the Wilson Sex Fantasy Questionnaire (reflecting sadomasochistic fantasies) and found a trend association (*p* < .07) between the self-reported paraphilic fantasies and non-right-handedness.

Because the androphilic and the paraphilic men have both shown elevated rates of non-right-handedness, these might seem to suggest that both groups match on this characteristic; however, the effect sizes thus far reported for these groups differ by an order of magnitude and would more reasonably be described as a large difference in this feature.

## Intelligence and Cognitive Profiles

IQ scores and profiles of relative cognitive strengths and weaknesses have long been used to provide insights into brain function. Although EEG, PET, fMRI, and other functional neuroimaging techniques have allowed researchers to observe brain activity more directly, neither cognitive neuropsychology nor its findings have become obsolete. The technological advancements have served largely to augment neuropsychological findings rather than to supplant them.

General cognitive ability, or IQ, has been repeatedly examined both in androphilic men and in paraphilic men. Overall, these reports suggested androphilic men to have the same or higher IQs than their gynephilic counterparts (for a review, see Weinrich, 1978), whereas paraphilic (mostly pedohebephilic) men have lower IQs than euphilic men (e.g., Blanchard et al., 2005; Cantor et al., 2004; see also Cantor, Blanchard, et al., 2005). Both claims need to be considered carefully, however, due to obvious ascertainment biases in recruiting samples of either group. In his re-analysis of prior samples of gay men, Weinrich (1978) noted, “As one moves from prisoners, to soldiers, to a clinical sample, to unmatched nonclinical samples, and finally to carefully matched nonclinical samples, the results move from highly mixed, to vaguely positive [gay men scoring higher in IQ], to significantly positive, to nearly ironclad” (p. 286). Despite that conclusion, it is more plausible that higher intelligence relates to one’s willingness to identify oneself as gay and to participate in research studies, rather than that higher intelligence relates to homosexuality per se.

Neuropsychological profiles—that is, patterns of relatively higher and relatively lower performance across a range of tests that tap into a variety of cognitive tasks—have long been used as an indirect method of quantifying the level of development or health of brain regions used in performing those tasks. Because several neuropsychological tests reveal reliable sex differences (e.g., women outperform men on average on certain verbal tasks and men outperform women on average on certain spatial tasks; Kimura, 1999), studies of androphilic men (and, in only very few studies, gynephilic women) have sought to ascertain whether there was a reversal in this pattern among homosexual teleiophiles. For several (but not all) sexually dimorphic tasks, this has indeed turned out to be the case in most studies (Cohen, 2002; Gladue, Beatty, Larson, & Staton, 1990; Hall & Kimura, 1995; Maylor et al., 2007; McCormick & Witelson, 1991; Neave, Menaged, & Weightman, 1999; Rahman, Abrahams, & Wilson, 2003; Rahman, Andersson, & Govier, 2005; Rahman & Koerting, 2008; Rahman & Wilson, 2003; Rahman, Wilson, & Abrahams, 2003, 2004; Sanders & Ross-Field, 1986a, b; Sanders & Wright, 1997; Wegesin, 1998a, b), but not all (e.g., Gladue & Bailey, 1995).

Neuropsychological profiles of the paraphilias have been ascertained largely from studies of sex offenders, including those diagnosed with sexual sadism (Hucker et al., 1988; Langevin et al., 1985), exhibitionism (Baker, 1985; Langevin, Lang, Wortzman, Frenzel, & Wright, 1989), and especially pedohebephilia (Bowden, 1987; Cantor et al., 2004; Cohen et al., 2002; Eastvold, Suchy, & Strassberg, 2011; Hucker et al., 1986; Jacobs, 1998; Langevin, Wortzman, Dickey, Wright, & Handy, 1988; Rubenstein, 1992; Suchy, Whittaker, Strassberg, & Eastvold, 2009). Other studies of sexual offenders committing the same types of offenses have also been published, but did not include any explicit method of ascertaining whether the subjects were genuinely paraphilic or engaged in the same behavior in service of some other motivator.

Considered together, these studies have not revealed any neuropsychological profile to be reliably associated either with any individual paraphilia or with paraphilias in general (for a complete review, see Blanchard, Cantor, & Robichaud, 2006). Studies of small samples have reported little, if any, group differences, whereas studies of large samples have reported deficiencies on nearly every neuropsychological test administered. Thus, the data suggest that paraphilic sex offenders manifest a general, but low- to moderate-sized, deficit in overall neuropsychological functioning.

## Neuroanatomy

Neuroanatomical investigations of androphilic men were conducted mostly in the 1990s, typically using as subjects men who had died of HIV/AIDS, dissecting specific structures within the hypothalamus, and focusing on structures already known to be sexually dimorphic. (That is, these studies pursued the brain-sex theory of homosexuality.) The studies of paraphilic (mostly pedohebephilic) men were conducted more recently, using MRI on living, otherwise healthy participants to quantify the whole brain. (That is, these studies were largely exploratory, requiring larger samples and more stringent statistical control.) Although both literatures are small and quite incomplete, the set of brain structures thus far associated with male androphilia do not overlap with the set of structures thus far associated with paraphilia. (No neuroanatomic studies of gynephilic or paraphilic women have yet been reported.)

The most widely discussed neuroanatomic difference between androphilic and gynephilic men was LeVay’s (1991) report that the third interstitial nucleus of the anterior hypothalamus (INAH-3) was smaller (i.e., shifted towards the female-typical direction) among androphiles. The same shift was subsequently found by Byne et al. (2001). (Although these latter authors described the difference as a “trend,” a one-tailed test of the directional hypothesis—that INAH-3 was smaller in androphilic men—would have met the conventional *p* < .05 criterion.) One study reported the suprachiasmatic nucleus of the hypothalamus to be denser and more voluminous in androphilic than gynephilic men (Swaab & Hoffman, 1990), but no attempt at replication has yet been reported. A lack of difference between androphilic and gynephilic men was reported for the mamillary bodies of the hypothalamus (Kruijver, Fernandez-Guasti, Fodor, Kraan, & Swaab, 2001).

Three studies have been conducted of pedophilic men, two comparing them with males recruited from the general community (Schiffer et al., 2007; Schiltz et al., 2007) and one comparing them with men who committed nonsexual offenses (Cantor et al., 2008). The Schiltz study, which focused on limbic structures, reported the hypothalamus (of the left hemisphere only) to be smaller among the pedophiles than among the controls; however, no study has yet described the morphology of individual nuclei within the hypothalamus.

In addition to the hypothalamic research, two white matter fiber bundles have been examined in androphilic men: the corpus callosum and the anterior commissure, both of which connect the left and right hemispheres of the brain, and both of which have been reported to be larger or denser in female than in male brains (Allen & Gorski, 1991, 1992; Driesen & Raz, 1995; Highley et al., 1999). Using MRIs of healthy subjects, Witelson et al. (2008) found androphilic men to be shifted towards the female-typical direction relative to gynephilic men. Allen and Gorski (1992) reported the anterior commissure to be thicker, also making it more female-typical, relative to gynephilic men. Lasco, Jordan, Edgar, Petito, and Byne (2002), however, reported finding no difference between androphilic and gynephilic men, but also failed to find any sex difference. In paraphilias research, Cantor et al. (2008) reported significantly lower density among pedophilic men in large spans of the superior occipitofrontal fasciculus (bilaterally) and the arcuate fasciculus (right hemisphere only). Although neither Schiltz et al. (2007) nor Schiffer et al. (2007) reported white matter differences, the Cantor study employed a much larger sample, thus applying greater statistical power. None of these three neuroanatomical studies of pedophilia detected any reliable differences in density in either the corpus callosum or the anterior commissure.

Both Schiltz et al. (2007) and Schiffer et al. (2007) reported significant differences in several other brain structures; however, none of those structures has yet been the subject of investigation by homosexuality researchers. Interestingly, two studies have implicated the bed nucleus of the stria terminalis (BNST) in a paraphilia (pedophilia in Schiltz et al., 2007; and autogynephilia in Zhou, Hofman, Gooren, & Swaab, 1995). Although the BNST finding in a probably autogynephilic sample might have been the result of their being anti-androgenic medications as part of the physical feminization process, Schiltz et al. (2007) did not report whether or what proportion of their subjects were also taking anti-androgenic medications, in their case, for the sex-drive reducing effects.

Thus, the literature has thus far identified nonoverlapping sets of anatomy to be related to male androphilia versus paraphilia; however, this conclusion is tentative at best: (1) The pedophilia studies all used MRI and lacked the resolution for providing reliable data on the very specific nuclei analyzed in the dissection studies of androphilic men. (2) The studies of the white matter in the pedophilic samples measured tissue density over entire structures whereas the androphilia studies focused on specific subportions.

## Conclusion

Overall, homosexuality and the paraphilias appear to share the features of onset and course (both homosexuality and paraphilia being life-long), but they appear to differ on sex ratio, fraternal birth order, handedness, IQ and cognitive profile, and neuroanatomy. Although there have been some reports on prevalence and on physical height, these literatures are not yet reliable enough to be informative. Thus, considered together, the existing data seem more consistent with the conclusion that homosexuality is a characteristic distinct from the paraphilias.

In considering the foregoing review, one should remember that the evidence is indirect: These correlates were not explored here because any of them is a *sine qua non* either of homosexuality or of any paraphilia. It is entirely possible that other, still unexplored, correlates are more central to the etiology of human sexual interests and that the correlates discussed in this article are merely tangential. Because only few paraphilic interests have received much scientific attention, it also remains possible that each paraphilia is associated with its own, novel set of correlates, and that homosexuality is no more novel in its profile of correlates than would be any other paraphilic interest. Thus, although homosexuality is probably better said to be distinct from the paraphilias, that conclusion is still quite tentative.
